# Hsa_circ_0023179 modulated the processes of proliferation, apoptosis, and EMT in non-small-cell lung cancer cells via the miR-615-5p/CDH3 axis

**DOI:** 10.17305/bb.2024.10944

**Published:** 2024-08-18

**Authors:** Qingkui Guo, Min Zheng, Chen Zhu, Bin Wu

**Affiliations:** 1 Department of Thoracic Surgery, Tongren Hospital, Shanghai Jiao Tong University School of Medicine, Shanghai, China

**Keywords:** Non-small-cell lung cancer (NSCLC), circ_0023179, Cadherin 3 (CDH3)

## Abstract

Circular RNA (circRNA) has been widely studied as a competitive endogenous RNA targeting microRNA (miRNA)/messenger RNA to regulate cancer progression. However, the regulatory mechanism of circ_0023179 in non-small-cell lung cancer (NSCLC) remains unclear. The expression levels of circ_0023179, miR-615-5p, and Cadherin 3 (CDH3) in NSCLC were detected using quantitative real-time polymerase chain reaction. The stability of circ_0023179 was verified using ribonuclease R enzyme, Actinomycin D and agarose gel electrophoresis. Colony formation and thymidine analog 5-ethynyl-2’-deoxyuridine assays were performed to examine proliferation changes in NSCLC cells. Western blot was used to assess the levels of CDH3 and epithelial–mesenchymal transition (EMT)-related marker proteins to evaluate EMT. Dual-luciferase reporter, RNA immunoprecipitation (RIP), and RNA pull-down assays were performed to explore the potential mechanisms of circ_0023179 in regulating NSCLC progression. Finally, the effects of circ_0023179 on NSCLC tumor growth in vivo were explored using a nude mouse subcutaneous tumor model. The results showed that the expression of circ_0023179 was remarkably higher in NSCLC tissues and cells, and it had a significant effect on NSCLC cell proliferation. Additionally, the knockdown of circ_0023179 significantly inhibited tumor growth in NSCLC mice. Mechanistically, circ_0023179 alleviated its inhibition of downstream CDH3 through the sponge-like adsorption of miR-615-5p. The downregulation of miR-615-5p and the upregulation of CDH3 mitigated the inhibitory effect of silencing circ_0023179 on NSCLC cell proliferation. In conclusion, silencing circ_0023179 inhibited NSCLC cell proliferation by targeting the miR-615-5p/CDH3 axis involved in NSCLC progression.

## Introduction

Lung cancer is a malignancy with high cancer-related mortality rates worldwide [1]. Non-small-cell lung cancer (NSCLC), a heterogeneous tumor, accounts for approximately 85% of all newly diagnosed lung cancers [2, 3]. Although treatments for NSCLC are being explored, the prognosis for patients remains poor. Therefore, identifying potential molecular targets is crucial for improving the diagnosis and prognosis of NSCLC.

Circular RNA (circRNA) is a unique, continuous, covalently closed-loop structure that lacks a 5′ cap and 3′ tail [4]. CircRNA is widely expressed in the cytoplasm and plays an integral role in the progression of various human cancers, including NSCLC [5, 6]. For instance, Circ_0000079 competitively binds to FXR1, inhibiting the formation of the FXR1-protein kinase C iota complex, thereby preventing cell invasion in NSCLC [7]. Wang and Li [8] demonstrated that silencing circ_0067934 inhibited NSCLC cell viability and impacted NSCLC prognosis. Previous studies have also indicated that the high expression of circ_0023179 in NSCLC is associated with histological type, TNM stage, lymph node metastasis, and distant metastasis. Compared with traditional tumor markers, circ_0023179 exhibits higher sensitivity and specificity, suggesting its potential as a biomarker for NSCLC [9]. However, the downstream regulatory mechanism of circ_0023179 in NSCLC progression remains unclear.

MicroRNAs (miRNAs) typically play a regulatory role in cancer development by targeting messenger RNAs (mRNAs) [10]. Studies have confirmed that miR-615-5p functions as a tumor suppressor in various cancers. For instance, miR-615-5p regulates the tumorigenicity of hepatocellular carcinoma (HCC) by negatively affecting oncogene levels, such as those of the insulin-like growth factor 1 receptor, serine hydroxymethyltransferase 2, and hydroxyacid oxidase 2 [11–13]. Additionally, miR-615-5p is regulated by circ_002144, which suppresses colorectal cancer metastasis by down-regulating La ribonucleoprotein 1 [14]. Furthermore, several studies have shown that circ_100146, circ_0004396, and LINC00324 act as sponges for miR-615-5p, thereby influencing the biological characteristics of NSCLC cells [15–17]. However, it remains unknown whether circ_0023179 regulates miR-615-5p to modulate NSCLC progression.

Cadherin 3 (CDH3), also known as P-cadherin, plays a key role in cell growth [18]. CDH3 is overexpressed in various cancers, including thyroid, pancreatic, and HCCs and is associated with a poor clinical prognosis [19–21]. However, its function in NSCLC is still unclear. In this study, we aimed to investigate the changes in the levels of circ_0023179, miR-615-5p, and CDH3 in NSCLC, as well as to elucidate their interactions using bioinformatics tools and relevant experimental methods. Specifically, this study aimed to demonstrate that circ_0023179 influences NSCLC progression through the miR-615-5p/CDH3 axis.

## Materials and methods

### Clinical tissue samples

A total of 45 NSCLC tissues and paired normal tissues were obtained from Tongren Hospital, Shanghai Jiao Tong University School of Medicine. All NSCLC patients provided informed consent. The study procedures were approved by the Ethics Committee of Tongren Hospital, Shanghai Jiao Tong University School of Medicine.

### Cell culture and cell transfection

Human NSCLC cell lines (A549, H520, H1299, and H1975) and human bronchial epithelial (HBE) cells (Cell Bank of Chinese Academy of Sciences, Shanghai, China) were maintained in Dulbecco’s Modified Eagle Medium (Invitrogen, Carlsbad, CA, USA) supplemented with 10% foetal bovine serum and 1% penicillin/streptomycin in a 5% carbon dioxide (CO_2_) incubator at 37 ^∘^C. The cells grown in the logarithmic proliferation phase were transfected with corresponding sh-NC, sh-circ_0023179, anti-miR-NC, anti-miR-615-5p, vector and CDH3 according to Lipofectamine 2000 reagent (Invitrogen) instructions. The plasmids and vectors were synthesized by GenePharma (Shanghai, China). Transfected cells were cultured for 48 h before being used for subsequent experiments.

### Quantitative real-time polymerase chain reaction (qRT-PCR)

Total cellular RNA was extracted using TRIzol reagent (Invitrogen), and complementary DNA (cDNA) was synthesized using the QuantiTect Reverse Transcription Kit (DXT-205314; Qiagen, Valencia, CA, USA). The cDNA was quantified using the QuantiTect SYBR Green PCR Kit (XT-204143; Qiagen) on an ABI7300-Fast qRT-PCR system (Applied Biosystems, Foster City, CA, USA). Relative quantification of the target genes was analyzed using the 2^-ΔΔCt^ method with glyceraldehyde-3-phosphate dehydrogenase (GAPDH) and U6 as internal references. Primer sequences: U6: F: 5′-CTCGCTTCGGCAGCACAT-3′; R: 5′-TTTGCGTGTCATCCTTGCG-3′; GAPDH: F: 5′-GTCTCCTCTGACTTCAACAGCG-3′; R: 5′-ACCACCCTGTTGCTGTAGCCAA-3′; circ_0023179: F: 5′-GGACACCAACATGATCGAGTCG3′; R: 5′CGCTCAATGCTGTGCAGATTCC-3′; miR-615-5p: F: 5′-TCCCCGGTGCTCGGA-3′; R: 5′-GAACATGTCTGCGTATCTC-3′; CDH3: F: 5′-CAGGTGCTGAACATCACGGACA-3′; R: 5′-CTTCAGGGACAAGACCACTGTG-3′.

### CircRNA validation

First, 5 µg of RNA was taken. Next, 2 µL of ribonuclease (RNase) R 10X Reaction Buffer, 0.5 µL of RNase R (10U, Sigma-Aldrich, St. Louis, MO, USA), and RNase-Free water were added to make a total volume of 20 µL. The mixture was centrifuged and digested at 37 ^∘^C for 30 min, then purified using a column. Transcription was inhibited by adding Actinomycin D (Act D; 2 mg/mL; Sigma-Aldrich) to the NSCLC cell culture medium for 0, 6, 12, and 24 h. The expression of circ_0023179 and LRP5 was then detected by qRT-PCR.

### Western blot analysis

Cells were lysed using radioimmunoprecipitation assay lysis buffer (Beyotime, Shanghai, China). Electrophoresis was performed with 12% sodium dodecyl-sulfate polyacrylamide gel electrophoresis, and the proteins were then transferred to polyvinylidene fluoride membranes (Millipore, Billerica, MA, USA). The membranes were incubated with primary antibodies (dilution at 1:1000, Abcam, Cambridge, MA, USA) against N-cad (ab76011), Snail1 (ab216347), E-cad (ab231303), CDH3 (ab242060), and GAPDH (ab8245) at 4 ^∘^C for 24 h. Secondary antibodies (1:5000 dilution) conjugated with horseradish peroxidase were applied for 2 h at 37 ^∘^C. Protein bands were detected by chemiluminescence using a Tanon 5200 chemiluminescence gel imaging system (Tennant, Shanghai, China).

### Colony formation assays

After cell digestion and centrifugation, the cells were counted. Next, 5 mL of medium was added to each well, which contained 1000 cells, and the cells were evenly distributed at the bottom of the six-well plate. The cells were incubated in a 5% CO_2_ incubator at 37 ^∘^C. The medium was changed every 3–5 days. After 5 days, the cell growth status was observed daily. Depending on the cells, fixation and staining began after 7–14 days when each colony was larger than 50 cells. Pictures were taken with a camera (Nikon, Tokyo, Japan), and ImageJ was used to analyze the results and count the number of colonies.

### EdU assays

The cells (1×10^5^ cells/well) were inoculated into 96-well plates and incubated at 37 ^∘^C for 24 h. After PBS washing, 10 µM EdU culture medium was added and incubated for 1 h in the dark. The cells were washed with PBS, fixed with 4% paraformaldehyde for 15 min at room temperature, and permeabilized with PBS containing 0.3% Triton X-100 (X100, Sigma-Aldrich) for 10 min. The Click reaction solution was incubated in the dark for 30 min, and the DAPI dye solution (D9542, Invitrogen) was then added to stain the nucleus for 10 min. After anti-quenching, the cells were observed with an IX73 fluorescent microscope (Olympus, Tokyo, Japan) at a magnification of 200×.

### RNA pull-down assays

The A549 and H1299 cells were transfected with biotin-labeled miR-615-5p (Bio-miR-615-5p) and a negative control (Bio-miR-NC), respectively. After 48 h, the cells were lysed, the supernatant was collected, and magnetic beads labeled with streptavidin were added and mixed. The levels of circ_0023179 in the RNA complexes bound to the beads were then measured by qRT-PCR.

### RIP assays

A Magna RNA immunoprecipitation (RIP) kit (Millipore) was used to enrich circ_0023179 and miRNA. The cells were lysed with RIP lysis buffer, and 100 µL of whole cell extract was treated with magnetic beads containing a circ_0023179 antibody at room temperature for 2 h. Subsequently, the samples were washed with RIP washing buffer and treated with proteinase K at 55 ^∘^C for 30 min to isolate RNA-protein complexes from the magnetic beads. qRT-PCR was used to detect target circRNA and miRNA.

### Dual-Luciferase reporter assays

Wild-type (WT) circ_0023179/CDH3 and mutant (MUT) circ_0023179/CDH3 were PCR-amplified and inserted into the pmirGLO reporter vector in the following order: circ_0023179_wt_, circ_0023179_mut_, CDH3 3′UTR_wt_, CDH3 3′UTR_mut_. Subsequently, circ_0023179_wt_ and miR-NC, circ_0023179_wt_ and miR-615-5p mimics, circ_0023179_mut_ and miR-NC, circ_0023179_mut_ and miR-615-5p mimics, CDH3 3′UTR_wt_ and miR-NC, CDH3 3′UTR_wt_ and miR-615-5p mimics, CDH3 3′UTR_mut_ and miR-NC, and CDH3 3′UTR_mut_ and miR-615-5p mimics were co-transfected into the A549 and H1299 cells, respectively, using Lipofectamine 2000. Cells were collected after 48 h of incubation, and luciferase activity was detected using the Dual-Luciferase Reporter Assay System (Promega, Madison, WI, USA).

A total of ten male BALB/c nude mice (aged four weeks) were obtained from Beijing Viton Lever Laboratory Animal Technology Co., Ltd. All mice were housed under specific pathogen-free conditions and maintained in a laminar flow cabinet. The mice were then divided into the sh-NC and sh-circ_0023179 groups (with five mice per group). A549-sh-NC and A549-sh-circ_0023179 cells were injected through the skin into the BALB/c nude mice. Tumor size was recorded weekly by measuring the tumor length and width, and the volume was calculated as follows: volume ═ (length × width^2^)/2. After four weeks, the nude mice were sacrificed, tumor tissues were collected and tumor volume was measured and weighed. All procedures were approved by the Ethics Committee of Tongren Hospital, Shanghai Jiao Tong University School of Medicine. The tumor tissues were divided into two parts: one part was fixed with 4% paraformaldehyde and paraffin-embedded to prepare tissue sections for immunohistochemical (IHC) staining of Ki-67, and the positive expression of Ki-67 was analyzed using ImageJ software. The other part was used to obtain RNA and related marker proteins from the tumor tissue.

### Ethical statement

The study procedures were approved by the Ethics Committee of Tongren Hospital, Shanghai Jiao Tong University School of Medicine.

### Statistical analysis

All experiments were independently repeated at least three times. Statistical analysis was performed using GraphPad Prism 5.0, and data are expressed as the mean ± standard deviation. Differences between multiple groups were analyzed using one-way analysis of variance. The Student’s *t*-test was used to compare differences between two independent groups. A *P* value < 0.05 was considered statistically significant.

## Results

### Circ_0023179 was stably hyper-expressed in NSCLC

**Figure 1.**
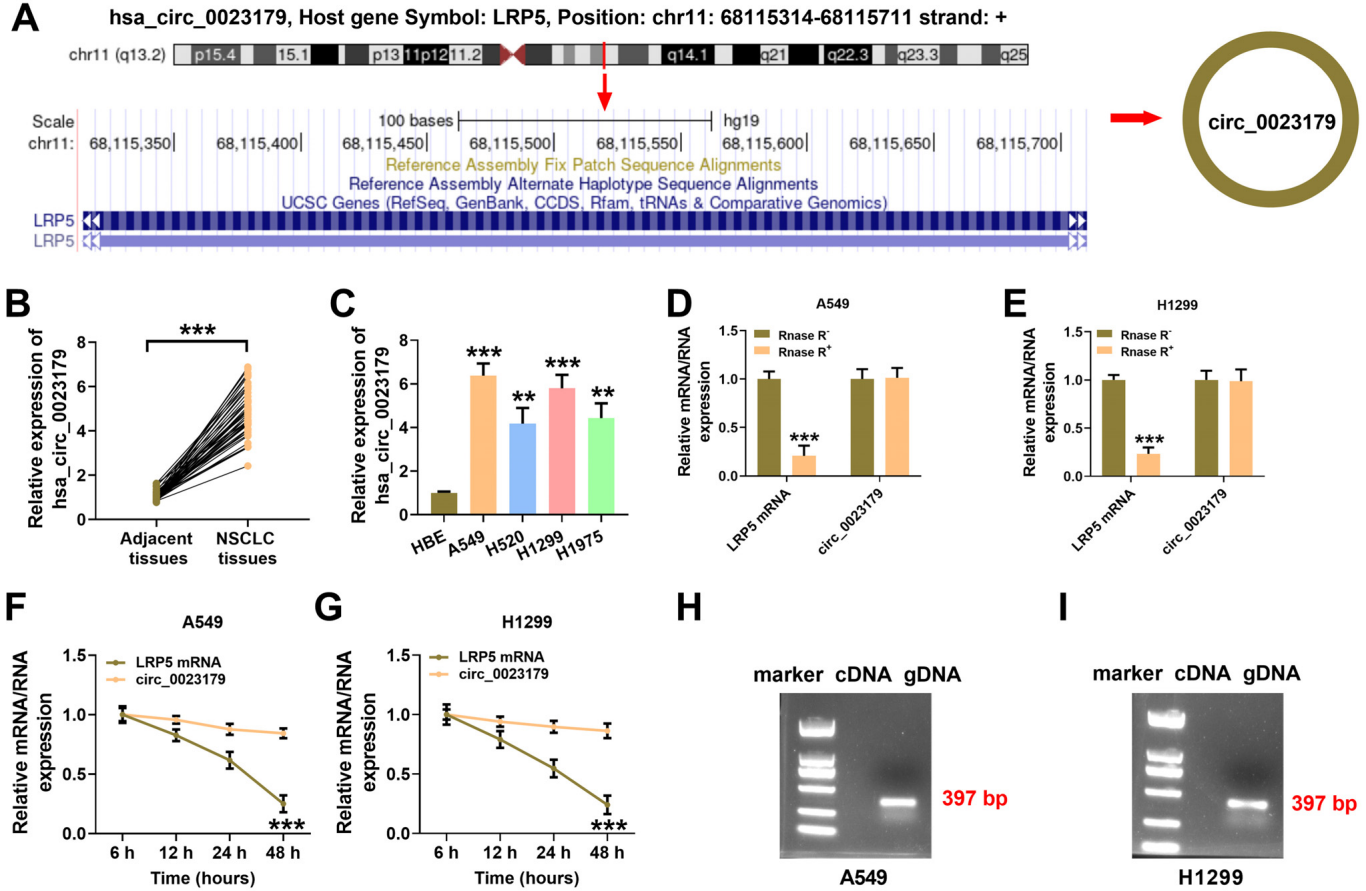
**Circ_0023179 was highly expressed in NSCLC.** (A) Schematic diagram of the structure of circ_0023179; (B) qRT-PCR was used to detect the expression of circ_0023179 in NSCLC tissues and paraneoplastic tissues; (C) qRT-PCR was used to detect the expression of circ_0023179 in HBE cells and four NSCLC cell lines (A549, H520, H1299, and H1975); (D and E) qRT-PCR was used to detect circ_0023179 and LRP5 in A549 and H1299 cells treated with RNase R; (F and G) qRT-PCR was used to detect circ_0023179 and LRP5 in A549 and H1299 cells treated with actinomycin D; (H and I) Nucleic acid electrophoresis was used to verify the PCR products of the cDNA and gDNA transcripts of circ_0023179. *P < 0.05, **P < 0.01, ***P < 0.001. NSCLC: Non-small-cell lung cancer; qRT-PCR: Quantitative real-time polymerase chain reaction; HBE: Human bronchial epithelial; cDNA: Complementary DNA.

Research has reported that LRP5 plays a role in regulating the prognosis of multiple cancers, including NSCLC [22, 23]. Furthermore, the action of circRNA is closely linked to the action of its corresponding mRNA, as circRNA is derived from the reverse splicing of the precursor mRNA. The reverse splice site of circ_0023179 in its corresponding mRNA, LRP5, and its loop structure are shown in Figure 1A. Circ_0023179 was further analyzed to confirm its high expression in NSCLC tissues (see Figure 1B). The expression of circ_0023179 in four NSCLC cell lines was measured, revealing that circ_0023179 was most highly expressed in the A549 and H1299 cells (see Figure 1C). Additionally, circ_0023179 was resistant to RNase R digestion, while LRP5 was largely digested by RNase R (see Figure 1D and 1E). Similarly, the Act D assays confirmed the stability of circ_0023179 (see Figure 1F and 1G). Subsequently, nucleic acid electrophoresis was used to verify the PCR products of the cDNA and gDNA of circ_0023179, with the 379 bp gDNA being detected (see Figure 1H and 1I).

### Knockdown of circ_0023179 inhibits NSCLC proliferation and epithelial–mesenchymal transition (EMT)

**Figure 2.**
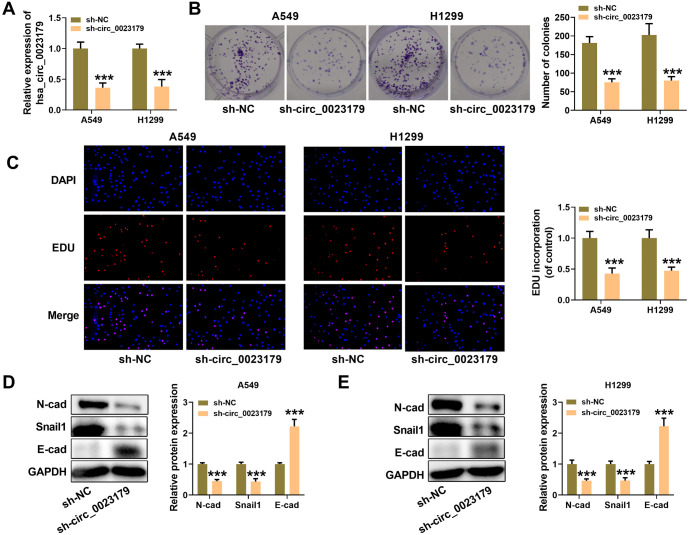
**Biological characteristics of circ_0023179 in NSCLC cells.** (A) qRT-PCR was used to verify the transfection efficiency of two stably transfected cell lines, A549-sh-circ_0023179 and H1299-sh-circ_0023179; (B) Colony formation assays were used to assess the effect of circ_0023179 knockdown on the proliferation ability of NSCLC cells; (C) EdU assays were used to evaluate the proliferation ability of two stably transfected cell lines, A549-sh-circ_0023179 and H1299-sh-circ_0023179; (D and E) Western blot assays were used to detect EMT-related proteins. *P < 0.05, **P < 0.01, ***P < 0.001. NSCLC: Non-small-cell lung cancer; qRT-PCR: Quantitative real-time polymerase chain reaction; EMT: Epithelial–mesenchymal transition.

Preliminary assays showed that circ_0023179 was optimally expressed in NSCLC cells. Consequently, circ_0023179 was selected for knockdown in two cell lines (A549-sh-circ_0023179 and H1299-sh-circ_0023179) to generate stable knockdown cell lines (see Figure 2A). Flat-plate clonogenesis assays revealed that sh-circ_0023179 decreased the number of clonal cells in NSCLC cells (see Figure 2B). Similarly, the EdU results showed that sh-circ_0023179 inhibited NSCLC cell proliferation (see Figure 2C). Additionally, after circ_0023179 knockdown, the expression of N-cad and Snail1 proteins decreased, while the expression of E-cad protein increased (see Figure 2D and 2E).

### Circ_0023179 sponge-like adsorption of MiR-615-5p

**Figure 3.**
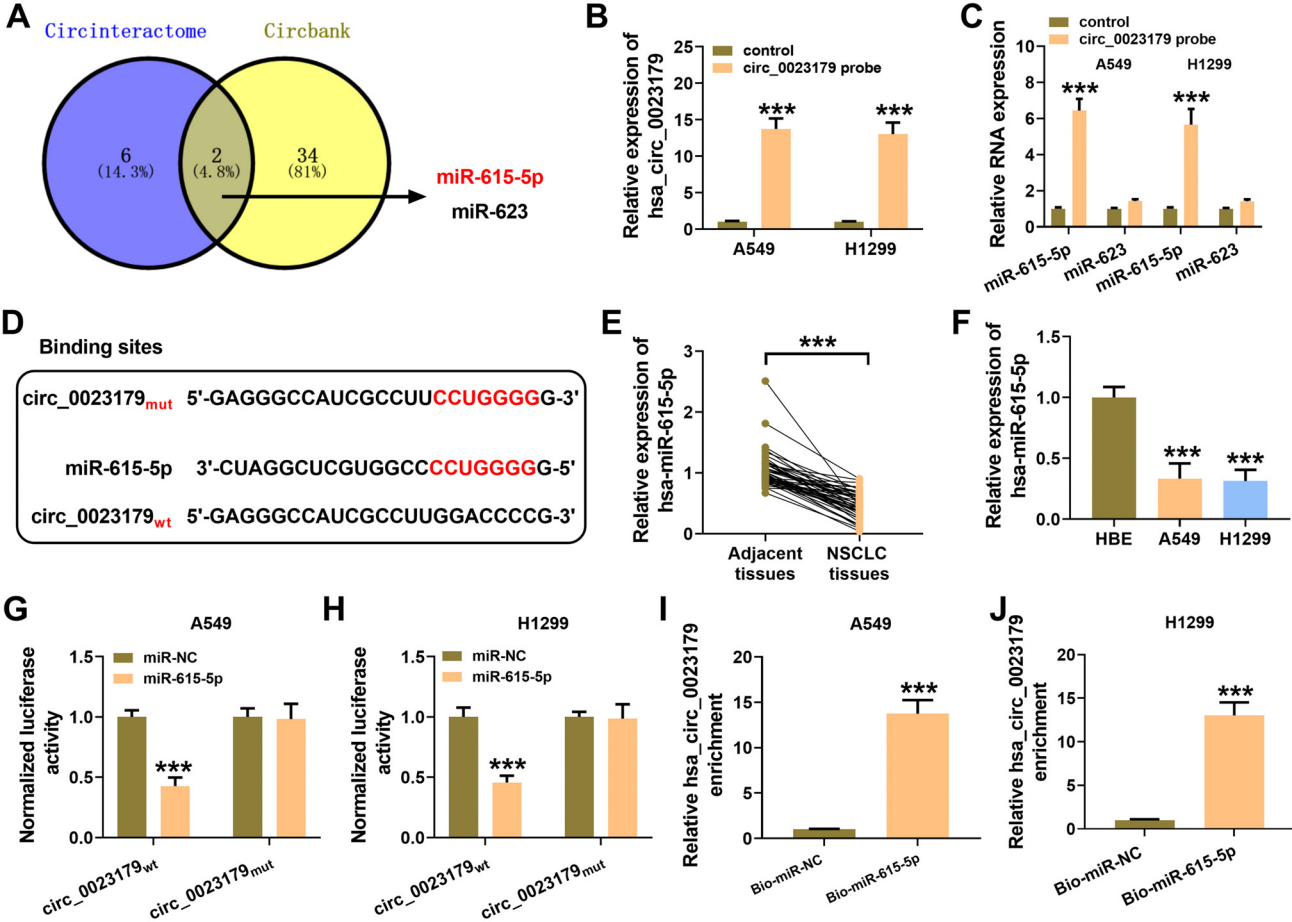
**circ_0023179 targeted miR-615-5p.** (A) The circInteractome and circbank biology websites were used to predict the target miRNAs for circ_0023179; (B and C) A549 and H1299 cells were examined using a circ_0023179 probe in RIP assays; (D) The binding site of circ_0023179 for miR-615-5p was predicted; (E) qRT-PCR was used to detect the expression of miR-615-5p in NSCLC tissues and paraneoplastic tissues; (F) qRT-PCR was used to detect the expression of miR-615-5p in the HBE, A549, and H1299 cells; (G and H) Dual-luciferase reporter assays were used to verify the binding of circ_0023179 and miR-615-5p; (I and J) RNA pull-down assays were used for the biotin-labeled miR-615-5p in A549 and H1299 cells. *P < 0.05, **P < 0.01, ***P < 0.001. miRNA: MicroRNA; RIP: RNA immunoprecipitation; qRT-PCR: Quantitative real-time polymerase chain reaction; NSCLC: Non-small-cell lung cancer.

Potential miRNAs predicted to interact with circ_0023179 using biological databases included miR-615-5p and miR-623 (Figure 3A). RIP was performed with a circ_0023179 probe in A549 and H1299 cells, revealing that miR-615-5p was significantly enriched (see Figure 3B and 3C). Figure 3D shows the predicted binding sites of circ_0023179 to miR-615-5p. MiR-615-5p was lowly expressed in NSCLC tissues and cells (see Figure 3E and 3F). Furthermore, miR-615-5p mimics were co-transfected with luciferase plasmids containing circ_0023179 WT and MUT sequences into NSCLC cells, and miR-615-5p was found to significantly reduce the luciferase activity of circ_0023179 WT but not circ_0023179 MUT (see Figure 3G and 3H). The miR-615-5p pull-down assays also showed that circ_0023179 was considerably enriched (see Figure 3I and 3J).

### MiR-615-5p inhibitor inverted the regulative effect of sh-circ_0023179 on NSCLC cells

**Figure 4.**
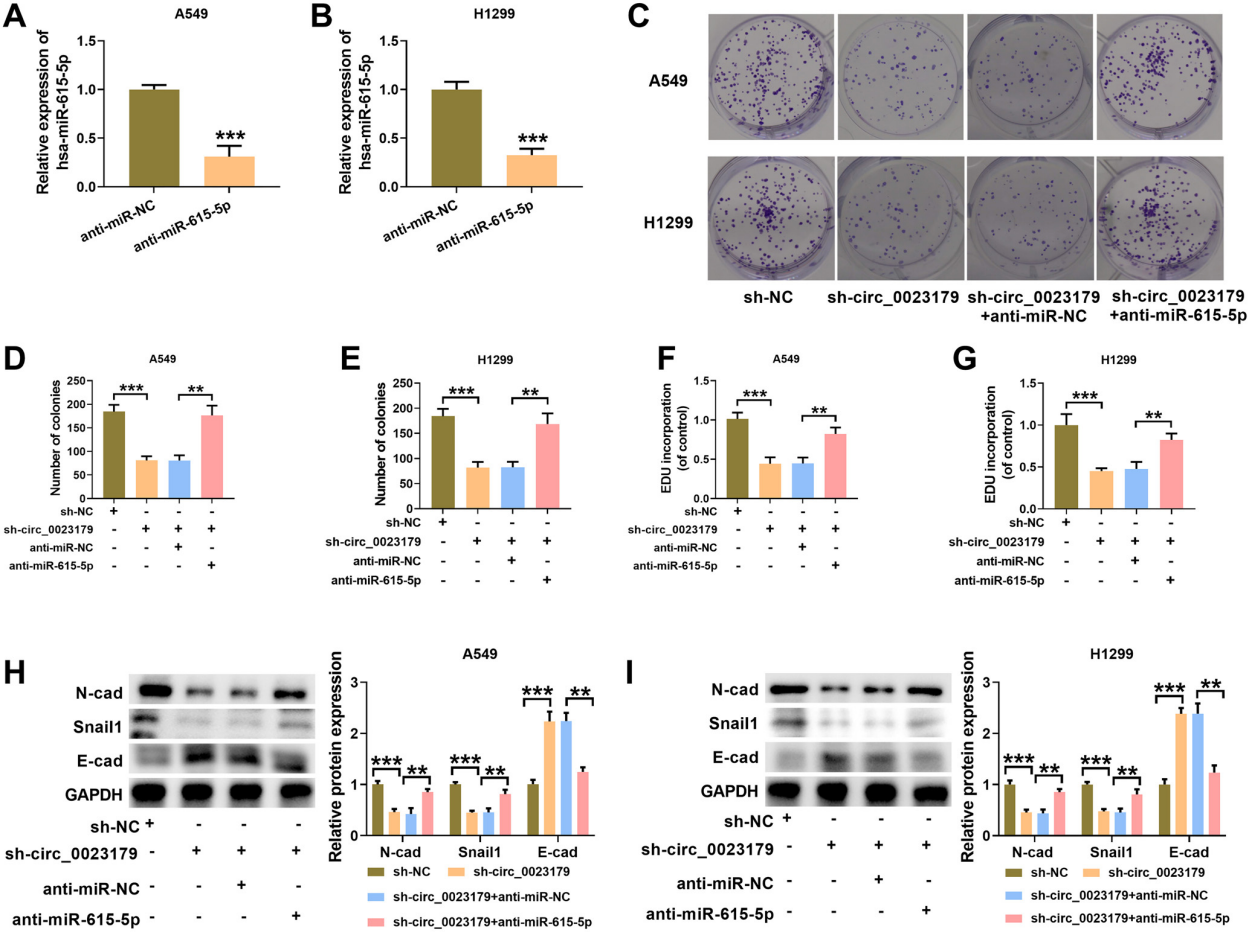
**Anti-miR-615-5p attenuated the inhibitory effect of sh-circ_0023179 on the proliferation of NSCLC cells.** (A and B) qRT-PCR was used to verify the knockdown efficiency of miR-615-5p in NSCLC cells; (C–E) Colony formation assays were used to detect the number of cell clones; (F and G) EdU assays were used to assess cell proliferation efficiency; (H and I) Western blot was used to detect EMT-related marker proteins. *P < 0.05, **P < 0.01, ***P < 0.001. NSCLC: Non-small-cell lung cancer; qRT-PCR: Quantitative real-time polymerase chain reaction; EMT: Epithelial–mesenchymal transition.

First, miR-615-5p was knocked down and introduced into A549 and H1299 cells. qRT-PCR was used to assess the knockdown efficiency (see Figure 4A and 4B). Next, anti-miR-615-5p was transfected into A549-sh-circ_0023179 and H1299-sh-circ_0023179 cell lines. Plate-clone formation and EdU assay results showed that anti-miR-615-5p promoted the proliferation of A549-sh-circ_0023179 and H1299-sh-circ_0023179 cells (see Figure 4C–4G). Additionally, the knockdown of miR-615-5p in A549-sh-circ_0023179 and H1299-sh-circ_0023179 cells increased the levels of EMT-related markers N-cad and Snail1, and decreased E-cad levels in both cell lines (see Figure 4H and 4I).

### MiR-615-5p targeted CDH3

**Figure 5.**
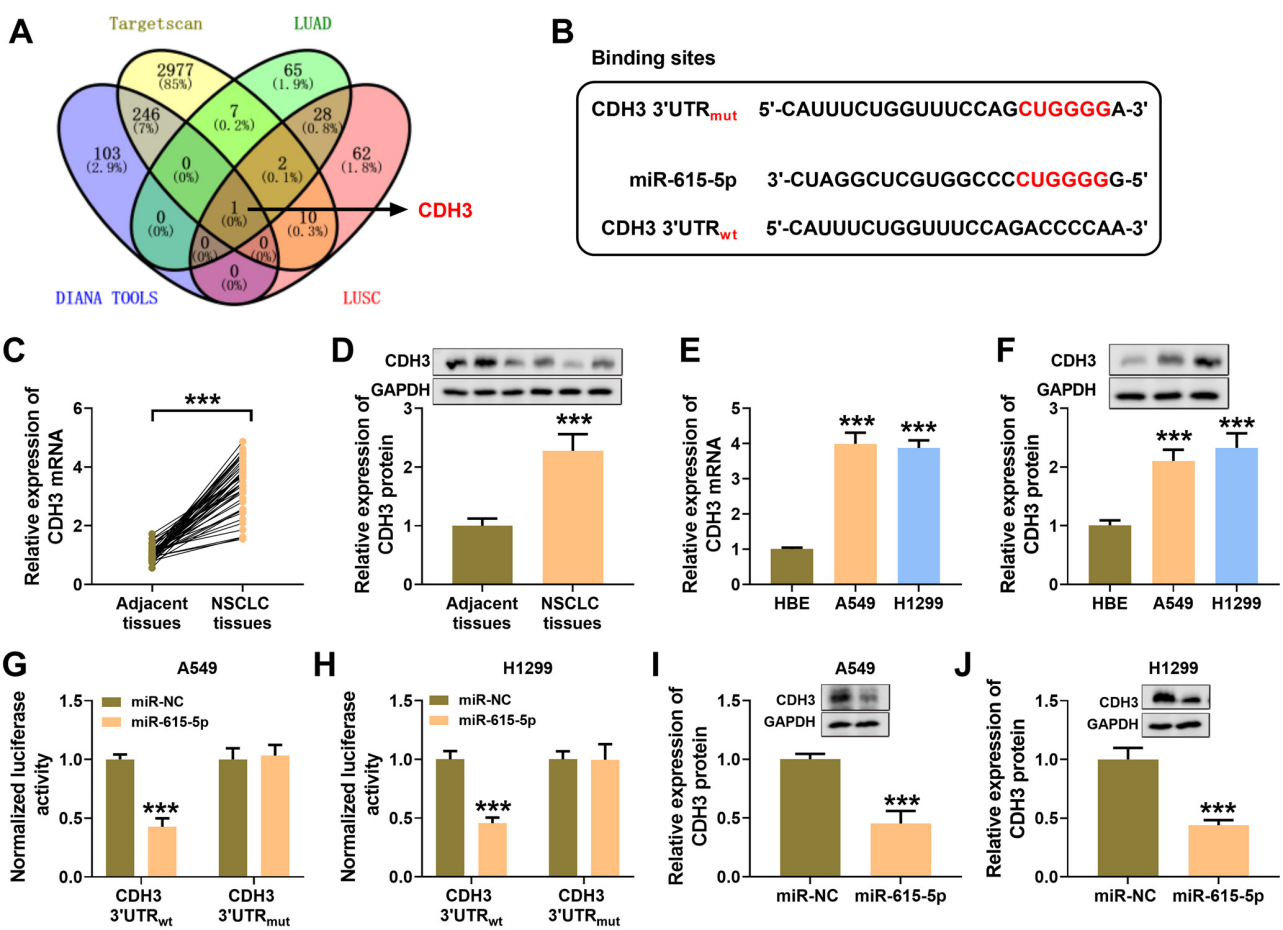
**CDH3 was a downstream gene of miR-615-5p.** (A) Four bioinformatic sites (i.e., targetscan, LUAD, DINAN TOOLS, and LUSC) were used to predict the potential target mRNA of miR-615-5p; (B) The binding sites of miR-615-5p to CDH3; (C) qRT-PCR was used to detect CDH3 expression in the paraneoplastic and NSCLC tissues; (D) Western blot was used to detect the expression of CDH3 protein in the paraneoplastic and NSCLC tissues; (E) qRT-PCR was used to detect the expression of CDH3 in HBE, A549, and H1299 cells; (F) Western blot was used to detect CDH3 protein expression in HBE, A549, and H1299 cells; (G and H) Dual-luciferase reporter assays were used to verify the binding of CDH3 mRNA and miR-615-5p; (I and J) Western blot assay was used to examine the effect of miR-615-5p on CDH3 protein expression in A549 and H1299 cells. *P < 0.05, **P < 0.01, ***P < 0.001. CDH3: Cadherin 3; mRNA: Messenger RNA; qRT-PCR: qRT-PCR: Quantitative real-time polymerase chain reaction; HBE: Human bronchial epithelial.

CDH3 was predicted to be a downstream target of miR-615-5p by four bioinformatics sites; the predicted binding sites for CDH3 and miR-615-5p are shown in Figure 5A and 5B. Notably, Figure 5C and 5D shows high expression of CDH3 in NSCLC tissues. Additionally, CDH3 expression was higher in NSCLC cells compared to HBE cells (see Figure 5E and 5F). The results showed that miR-615-5p mimics could bind the CDH3 3′UTR sequence, leading to decreased luciferase activity (see Figure 5G and 5H). The overexpression of miR-615-5p suppressed CDH3 expression in NSCLC cells (see Figure 5I and 5J).

### Overexpression of CDH3 attenuated the proliferation inhibitory effect of circ_0023179 knockdown on NSCLC cells

**Figure 6.**
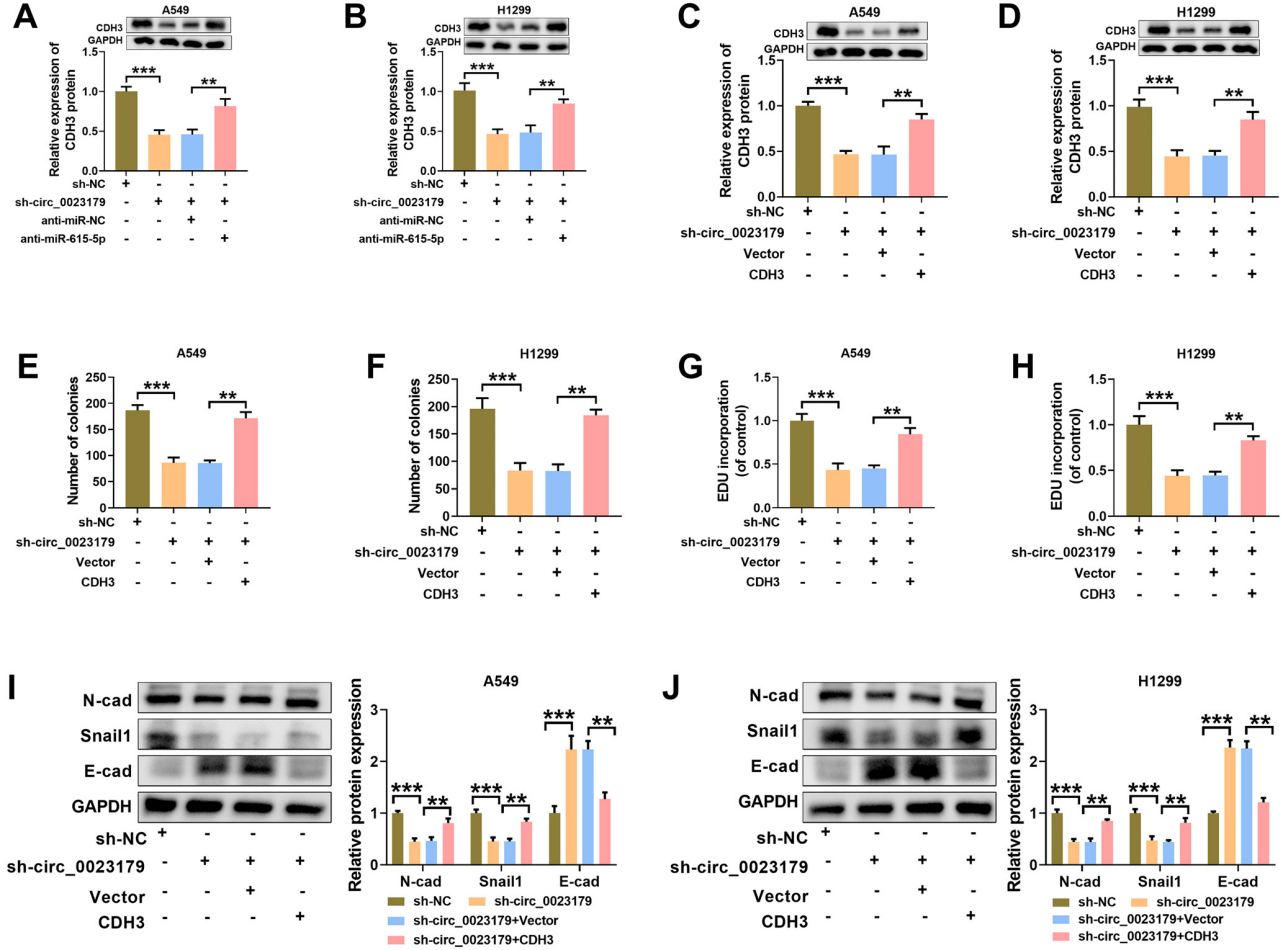
**The overexpression of CDH3 improved the inhibitory effect of sh-circ_0023179 on the proliferation of NSCLC cells.** (A and B) Circ_0023179 and miR-615-5p were both knocked down in A549 and H1299 cells, and western blot was used to detect CDH3 protein expression; (C and D) Western blot was used to detect CDH3 protein expression after the knockdown of circ_0023179 and the overexpression of CDH3 in A549 and H1299 cells; (E and F) Colony formation assays were used to detect the number of cell clones; (G and H) EdU assays were used to examine cell proliferation efficiency; (I and J) Western blot assays were used to detect the EMT-related marker proteins. *P < 0.05, **P < 0.01, ***P < 0.001. CDH3: Cadherin 3; NSCLC: Non-small-cell lung cancer; EMT: Epithelial–mesenchymal transition.

The knockdown of miR-615-5p in A549-sh-circ_0023179 and H1299-sh-circ_0023179 cells significantly increased CDH3 expression (see Figure 6A and 6B). Similarly, CDH3 levels were restored in A549-sh-circ_0023179 and H1299-sh-circ_0023179 cells by the overexpression of CDH3 (see Figure 6C and 6D). Additionally, the effect of sh-circ_0023179 on NSCLC cell growth was significantly mitigated by the overexpression of CDH3 (see Figure 6E–6H). The detection of EMT-related marker proteins revealed that the effects of silencing circ_0023179 on N-cad, Snail1, and E-cad proteins in NSCLC cells were reversed by the overexpression of CDH3 (see Figure 6I and 6J).

### Sh-Circ_0023179 inhibited NSCLC tumor growth

**Figure 7.**
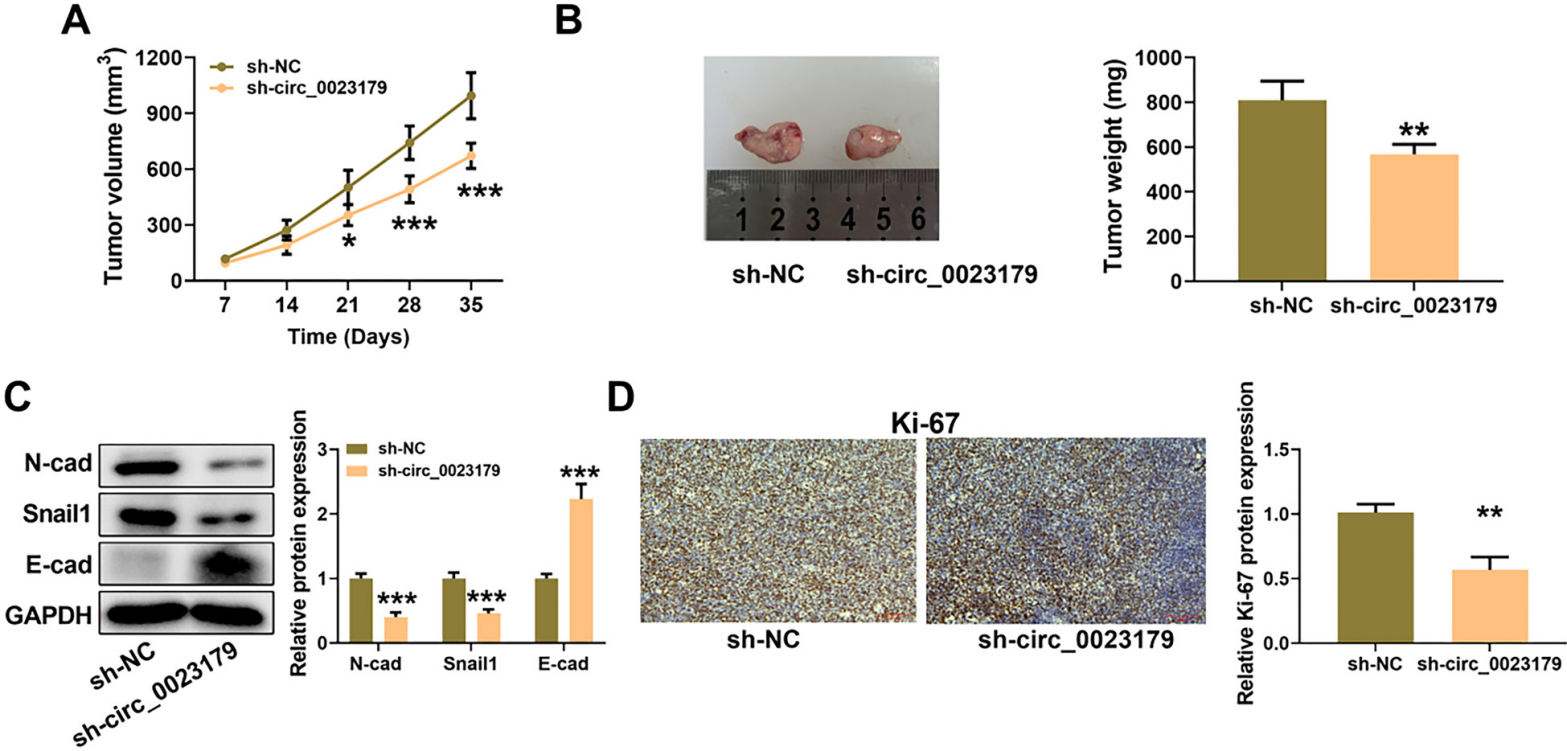
**sh-circ_0023179 inhibited NSCLC tumor growth the A549-sh-circ_0023179 cells were inoculated subcutaneously in nude mice.** (A) Tumor size was measured once a week; (B) Tumors were weighed once a week; (C) Western blot was used to detect EMT-related protein expression in tumor tissues; (D) IHC staining was used to detect the Ki-67 protein index. *P < 0.05, **P < 0.01, ***P < 0.001. NSCLC: Non-small-cell lung cancer; EMT: Epithelial–mesenchymal transition; IHC: Immunohistochemical.

In addition to in vitro assays, A549-sh-circ_0023179 cells were inoculated subcutaneously in nude mice to construct a nude mouse subcutaneous tumor model for in vivo experiments. The results showed that sh-circ_0023179 reduced tumor volume and weight in NSCLC (see Figure 7A and 7B). Western blot assay results showed that silencing circ_0023179 inhibited N-cad and Snail1 protein levels and upregulated E-cad protein content in the tumor tissues (see Figure 7C). IHC assay results showed that sh-circ_0023179 markedly reduced Ki-67 content in tumors (see Figure 7D).

## Discussion

In this study, the effects of circ_0023179 on NSCLC were investigated both in vitro and in vivo. Circ_0023179 regulates the proliferation, apoptosis, and EMT processes of NSCLC cells through the miR-615-5p/CDH3 axis. NSCLC is a malignant tumor, and although existing treatments have shown some efficacy, patient prognosis remains poor [24]. The study identified circ_0023179 as a potential biomarker for NSCLC [9].

Recent research has shown that circRNAs are robust against RNases and are present in various tissues and body fluids, resulting in a longer half-life and greater stability. This stability suggests that circRNAs may serve as diagnostic or predictive biomarkers for various cancers [25]. For example, serum levels of circ-FAF1 and circ-ELP3 have been proposed as novel potential biomarkers for breast cancer diagnosis [26]. In gastric cancer patients, a low level of hsa:circ_0001811 indicates a better prognosis [27]. Additionally, circRNAs have been implicated in cancer genesis and pathogenesis; for instance, hsa:circ_0005273 has been shown to promote breast cancer tumorigenesis by sponging miR-200a-3p to upregulate YAP1, thereby affecting the Hippo pathway [28]. In this study, the knockdown of circ_0023179 in NSCLC inhibited the proliferative capacity of NSCLC cells by suppressing the EMT process.

Available data suggest that circRNAs may play a crucial role in the onset and progression of various human diseases [29]. To date, most research on circRNAs has focused on their function as miRNA sponges or competitive endogenous RNAs [25]. CircRNAs containing miRNA response elements are primarily located in the cytoplasm and can negate the inhibitory effects of miRNAs on downstream targets by binding to complementary miRNA sequences [30]. This study investigated whether miR-615-5p binds to circ_0023179. Previous studies have shown that miR-615-5p functions as a tumor suppressor in several cancers, including HCC, prostate cancer, and nasopharyngeal carcinoma [12, 31, 32]. In this study, miR-615-5p was found to be lowly expressed in NSCLC. The knockdown of circ_0023179 led to elevated miR-615-5p levels, which reversed the inhibitory effect of sh-circ_0023179 on NSCLC cell proliferation, suggesting that miR-615-5p may also suppress cancer cell proliferation in NSCLC.

CircRNAs function by adsorbing and degrading miRNAs to increase mRNA levels of target genes, binding and regulating protein functions, or influencing epigenetic modifications, thereby directly regulating the expression of homologous genes [29]. In this study, we identified CDH3 as a downstream target of miR-615-5p through multiple databases. CDH3, a member of the cadherin family, was originally discovered in 1986 by Nose and Takeichi [33] in mouse embryonic development. This family constitutes an essential category of intercellular adhesion molecules involved in regulating cell polarity and differentiation, contributing to the maintenance of tissue homeostasis [34–36]. In normal human tissues, CDH3 expression is low, detected primarily in the thymus and fetal brain. However, high expression levels of CDH3 have been observed in various human tumor tissues [37]. Research has also shown that CDH3 promotes tumorigenesis in gastric cancer, pancreatic cancer, and breast cancer, while it inhibits tumorigenesis in HCC and melanoma [38–40]. Our results demonstrated that the knockdown of circ_0023179 increased miR-615-5p expression and decreased CDH3 expression, suggesting that circ_0023179 promotes the EMT process in NSCLC cells through the miR-615-5p/CDH3 axis, thereby enhancing cell proliferation.

## Conclusion

This study revealed that circ_0023179 increases CDH3 levels by sponging miR-615-5p, facilitating the EMT process and enhancing cell proliferation in NSCLC cells. These findings suggest that circ_0023179 could be a strategic target for the prospective diagnosis and treatment of NSCLC.

## Data Availability

The data supporting the findings of this study can be obtained from the corresponding author, upon request.
